# Peritonitis outcomes in patients with HIV and end-stage renal failure on peritoneal dialysis: a prospective cohort study

**DOI:** 10.1186/s12882-017-0466-0

**Published:** 2017-02-03

**Authors:** Kwazi C. Z. Ndlovu, Wilbert Sibanda, Alain Assounga

**Affiliations:** 1 Inkosi Albert Luthuli Central Hospital, Durban, South Africa; 2 Department of Nephrology, University of KwaZulu-Natal, P/Bag X7, Congella, Durban, 4013 South Africa; 3 School of Nursing and Public Health, University of KwaZulu-Natal, Durban, South Africa

**Keywords:** Continuous ambulatory peritoneal dialysis (CAPD), End-stage renal disease (ESRD), HIV, Peritonitis, Infection, Technique failure, Catheter failure

## Abstract

**Background:**

Few studies have investigated the management of human immunodeficiency virus (HIV)-associated end-stage renal failure particularly in low-resource settings with limited access to renal replacement therapy. We aimed to evaluate the effects of HIV infection on continuous ambulatory peritoneal dialysis (CAPD)-associated peritonitis outcomes and technique failure in highly active antiretroviral therapy (HAART)-treated HIV-positive CAPD populations.

**Methods:**

We conducted a single-center prospective cohort study of consecutive incident CAPD patients recruited from two hospitals in Durban, South Africa from September 2012-February 2015. Seventy HIV-negative and 70 HIV-positive end-stage renal failure patients were followed monthly for 18 months at a central renal clinic. Primary outcomes of peritonitis and catheter failure were assessed for the first 18 months of CAPD therapy. We assessed risk factors for peritonitis and catheter failure using Cox regression survival analysis.

**Results:**

The HIV-positive cohort had a significantly increased rate of peritonitis compared to the HIV-negative cohort (1.86 vs. 0.76 episodes/person-years, respectively; hazard ratio [HR], 2.41; 95% confidence interval [CI], 1.69–3.45, *P* < 0.001). When the baseline CD4 count was below 200 cells/μL, the peritonitis rate rose to 3.69 episodes/person-years (HR 4.54, 95% CI 2.35–8.76, *P* < 0.001), while a baseline CD4 count above 350 cells/μL was associated with a peritonitis rate of 1.60 episodes/person-years (HR 2.10, CI 1.39–3.15, *P* = 0.001). HIV was associated with increased hazards of peritonitis *relapse* (HR, 3.88; CI, 1.37–10.94; *P* = 0.010). Independent predictors associated with increased peritonitis risk were HIV (HR, 1.84; CI, 1.07–3.16; *P* = 0.027), diabetes (HR, 2.09; CI, 1.09–4.03; *P* = 0.027) and a baseline CD4 count < 200 cells/μL (HR, 3.28; CI, 1.42–7.61; *P* = 0.006). Catheter failure rates were 0.34 (HIV-positive cohort) and 0.24 (HIV-negative cohort) episodes/person-years (HR, 1.42; 95% CI, 0.73–2.73; *P* = 0.299). Peritonitis (HR, 14.47; CI, 2.79–75.00; *P* = 0.001), average hemoglobin concentrations (HR, 0.75; CI, 0.59–0.95; *P* = 0.016), and average serum C-reactive protein levels were independent predictors of catheter failure.

**Conclusions:**

HIV infection in end-stage renal disease patients managed by CAPD was associated with increased peritonitis risk; however, HIV infection did not increase the risk for CAPD catheter failure rate at 18 months.

## Background

Continuous ambulatory peritoneal dialysis (CAPD) is the dialysis modality of choice for many patients with end-stage renal disease (ESRD) and a cost-effective option easily implemented in low-resource settings [1–3]. However, peritonitis presents an ongoing challenge and is a major cause of technical failure [4–6], particularly under poor socioeconomic conditions and in immunocompromised patients [7, 8]. Considerable advancement has been made in CAPD management over the last decades leading to a substantial decrease in peritonitis rates, with as few as 1 case/51 patient–months reported by some authors [9]. However, peritonitis remains an important factor influencing CAPD-associated morbidity and mortality, and certain organisms, such as fungi and Gram-negative bacteria, are associated with worse outcomes [10–12]. Although reports are inconsistent, some of the factors associated with increased peritonitis risks are age, race, sex, comorbidities (diabetes, human immunodeficiency virus [HIV]), socioeconomic status, smoking, higher body mass index (BMI), malnutrition and chronic inflammation [4, 8, 13–16].

HIV infection presents a unique challenge in patients with ESRD managed with CAPD. As HIV impairs local host defense mechanisms [16], the risk of peritonitis in this population may be influenced by the adequacy of viral control and the patient’s immunologic state [8, 17]. Furthermore, the protein and amino acid losses frequently observed in CAPD may aggravate the malnutrition and hypoalbuminemia common in HIV infection, which can further compound the risk of peritonitis [18–20]. The rates of non-communicable diseases such as chronic kidney diseases (CKD) among HIV-positive populations are expected to rise significantly, as highly active antiretroviral therapy (HAART) becomes widely accessible, and life expectancy improves [21]. However, in economically disadvantaged regions such as sub-Saharan Africa where the HIV population is disproportionately concentrated, only a small percentage of those in need are expected to have access to renal replacement therapy [21–23]. CAPD is a relatively inexpensive, easily learned, and readily implemented dialysis option that does not require complex equipment [1–3, 18]. As such, it is particularly well suited as a home dialysis modality in regions where dialysis facilities are limited. However, peritonitis may complicate the use of CAPD in patients with ESRD and HIV. This study aimed to evaluate the effects of HIV infection on CAPD-associated peritonitis rates and outcomes, and to assess risk factors associated with the development of peritonitis and technique failure in HAART-treated HIV-positive CAPD populations.

## Methods

The study protocol was approved by the University of KwaZulu-Natal Biomedical Research Ethics Committee (BE 187/11), and research was conducted in accordance with the principles of the Declaration of Helsinki. All participants provided written informed consent prior to study enrollment.

### Sites

We recruited patients for a prospective cohort study from two hospitals in Durban, South Africa between September 2012 and February 2015. King Edward VIII Hospital (KEH) is a 799-bed regional referral center with limited specialist services. Inkosi Albert Luthuli Central Hospital (IALCH) is an 846-bed specialist referral hospital for KwaZulu-Natal province and covers a catchment area of approximately 10 million people. The renal unit based in IALCH offers CAPD (total patient population of 220), hemodialysis (150 patients), and transplantation services.

### Study population

We enrolled 70 HIV-negative and 70 HIV-positive patients with end-stage renal failure who underwent dialysis with a newly inserted double-cuffed coiled Tenckhoff catheter at the two hospitals. Patients with incident CAPD aged 18–60 years were consecutively recruited soon after Tenckhoff insertion until each cohort reached the 70-patient target. Peritonitis rate differentials reported by previous similar studies were used to calculate the sample size required to achieve a power of 80% and an α error probability of 0.05 [8]. The HIV infection status was determined by two 4^th^ generation HIV enzyme-linked immunosorbent assays (ELISA) performed by the South African National Health Laboratory Service (NHLS) before enrollment, screening for HIV performed using a HIV Ag/Ab Combo (CHIV) assay (ADVIA Centaur® XP, Siemens Healthcare Diagnostics, Tarrytown, NY, USA) and confirmation using HIV Combi and HIV Combi PT assays (Cobas e601, Roche Diagnostics, Mannheim, Germany). HAART management was left to the discretion of the local clinic. Tenckhoff catheter insertion was performed by experienced surgeons by laparoscopy (66 HIV-negative and 35 HIV-positive patients) at IALCH, and by trained nephrologists percutaneously at KEH (4 HIV-negative and 20 HIV-positive patients) and IALCH (15 HIV-positive patients). All CAPD patients utilized Y-sets, twin-bag systems, and conventional peritoneal dialysis (PD) solutions (Dianeal 1.5, 2.5, or 4.25% dextrose, icodextrin, or amino acid-based solutions; Baxter Healthcare, Deerfield, IL, US). They were trained predominantly as outpatients by the same nursing team, and generally performed four exchanges per day.

### Enrollment and follow-up

On enrollment, demographic, clinical, and biochemical data were recorded. All patients were followed-up at a central renal clinic at IALCH monthly for 18 months or until the endpoints of catheter removal or death. At each follow-up, vital signs, clinical assessment, anthropometric measurements, and phlebotomy for biochemical tests were done by the research team, and details of peritonitis events and hospital admissions in the intervening period were recorded on predefined questionnaires. Laboratory tests for full blood count, C-reactive protein (CRP), and serum urea, creatinine, electrolytes, albumin, and ferritin were performed by the NHLS, and results were periodically retrieved from the IALCH electronic results database.

### Peritonitis episodes

A peritonitis episode was defined as a clinical presentation with a cloudy effluent or abdominal pain associated with an effluent white blood cell count (WCC) of more than 100 cells/μL or a positive PD effluent culture. The diagnosis of peritonitis was made by the CAPD nurse and the attending physician. The attending physician decided whether to manage the case on an inpatient or outpatient basis depending on the severity of the clinical presentation. All patients initially received intraperitoneal vancomycin and amikacin empirically, and further therapy was modified according to culture results. Treatment duration was typically two weeks unless extended to three weeks by the attending physician due to the cultured organism or response to treatment. Episodes of peritonitis were recorded on predefined questionnaires during monthly clinic visits along with the date of presentation, whether treated as inpatient or outpatient, presenting PD WCC, and the culture result retrieved from the hospital electronic record. PD effluent WCCs were manually assessed using a 40X microscope, and PD effluent culturing was performed by the NHLS microbiology department using standard culturing techniques.

Peritonitis-associated hospital admission was defined as an admission for which peritonitis was cited as one of the indications or where peritonitis was diagnosed during the admission. The hospitalization episodes were recorded on the predefined questionnaire with the date of admission and discharge, indications for, and outcome of the admission. Catheter removal occurring during a peritonitis-associated admission episode was recorded as being related to peritonitis.

Multiple peritonitis episodes were classified as *relapsing* if occurring within 4 weeks of completion of treatment for a prior episode with the same organism or one sterile episode, *recurrent* if occurring within 4 weeks of completion of treatment for a prior episode with a different organism, or *repeat* if occurring more than 4 weeks after completion of treatment for the prior episode [24].

### Endpoints

All Tenckhoff catheters were removed at IALCH. The indications for removal and the corresponding date were recorded as study endpoints. Technique failure was defined as catheter removal due to catheter malfunction or infection. The in-hospital mortality dates at IALCH and certified causes of death were recorded. Deaths occurring outside IALCH were recorded as home deaths, and the corresponding details were obtained via telephone interviews with the participants’ relatives.

### Statistical analysis

Continuous variables are expressed as mean ± standard deviation or medians (interquartile range [IQR]) and were compared using the Student’s *t*-test or Wilcoxon-Mann-Whitney test as appropriate. Proportions and categorical variables were compared using Pearson’s chi-square test or Fisher’s exact test as appropriate. Survival estimates were computed using the Kaplan–Meier method, and the log-rank test was used to compare survival curves. Univariate Cox regression survival analysis was used to estimate the association between HIV, associated subgroups, and various risk factors for outcome variables. Multivariable Cox regression analysis was used to identify independent predictors of survival. All analyses were performed using Stata Statistical Software, Release 13 (StataCorp, College Station, Texas, US). The level of significance was set at *P* < 0.05.

## Results

### Patient characteristics

The mean patient age was 39.1 ± 11.7 (HIV-negative) and 37.0 ± 9.4 (HIV-positive) years with women accounting for 42.9 and 52.9% of the two cohorts, respectively. All patients (100%) in the HIV-positive cohort were of African ethnicity compared to 84.3% in the HIV-negative cohort (*P* = 0.003). Fifty-one percent of HIV-positive patients were either newly diagnosed with HIV or had recently started HAART (less than six months before insertion of the Tenckhoff catheter). However, 57.1% of HIV-positive patients had a suppressed viral load (<150 copies/mL, hospital laboratory assay limit) at the time of enrollment. While the median baseline viral load was 4230 copies/mL (IQR, 903–91,143) for patients with detectable viral loads, the median dropped below the detectable limit (IQR <150–2990) when including patients with undetectable viral loads. Twenty-one percent of HIV-positive patients had CD4 counts >500 cells/μL, 20.0% had <200 cells/μL, and the remainder (58.6%) had 200–500 cells/μL. Other details of the study population are outlined in Table 1 and were previously described in our report on 1-year outcomes [25].

**Table 1:** Baseline characteristics

Variable	HIV-Negative (*n* = 70)	HIV-Positive (*n* = 70)	*P* value
Age (mean ± SD)	39.1 ± 11.7	37.0 ± 9.4	0.247^a^
Weight, kg (mean ± SD)	68.9 ± 12.6	66.1 ± 13.7	0.213^a^
Body mass index (mean ± SD)	25.1 ± 4.7	24.5 ± 5.4	0.436^a^
Waist circumference, cm (mean ± SD)	90.5 ± 10.9	90.0 ± 11.5	0.822^a^
Sex			
Female, *n* (%)	30 (42.9)	37 (52.9)	0.236^b^
Ethnicity			
African, *n* (%)	59 (84.3)	70 (100.0)	0.001^c^
Indian, *n* (%)	9 (12.9)	0 (0.0)	
Mixed ethnicity, *n* (%)	2 (2.9)	0 (0.0)	
Hypertension, *n* (%)	63 (90.0)	52 (74.3)	0.015^b^
Diabetes, *n* (%)	4 (5.7)	7 (10.0)	0.532^c^
SLE, *n* (%)	4 (5.7)	1 (1.4)	0.366 ^c^
Hepatitis B, *n* (%)	7 (10.0)	8 (12.1)	0.737^b^
Primary residence			
City, *n* (%)	14 (20.0)	6 (8.6)	0.113^b^
Township^e^, *n* (%)	30 (42.8)	39 (55.7)	
Rural, *n* (%)	23 (32.9)	24 (34.3)	
Education level			
Primary school, *n* (%)	15 (21.4)	13 (18.6)	0.710^b^
High school, *n* (%)	32 (45.7)	31 (44.3)	
Post-grade 12, *n* (%)	20 (28.6)	25 (35.7)	
Employment history			
Unemployed, *n* (%)	50 (71.4)	53 (75.7)	0.766^b^
Employed, *n* (%)	17 (24.3)	16 (22.9)	
Smoking (currently)	4 (5.71)	7 (10.0)	0.532^c^
Tenckhoff catheter insertion method			
Laparoscopic, *n* (%)	66 (94.3)	35 (50.0)	<0.001^c^
Percutaneous, *n* (%)	4 (5.71)	35 (50.0)	
Hemoglobin, g/dL (mean ± SD)	9.60 ± 2.01	8.96 ± 1.61	0.041^a^
Albumin, g/L (mean ± SD)	35.3 ± 6.7	31.0 ± 6.6	<0.001^a^
eGFR, ml/min/1.73 m^2^(mean ± SD)	7.0 ± 3.6	7.1 ± 4.6	0.935^a^
Creatinine (μmol/l), median (IQR)	736.5 (542–974)	718 (598–888)	0.917^d^
CRP (mg/L), median (IQR)	19 (6–35)	56.5 (21–108)	<0.001^d^
ESR (mm/hr), median (IQR)	49 (29–66)	88 (50–129)	<0.001^d^
Ferritin (μg/L), median (IQR)	642 (370–1049)	593 (381–973)	0.858^d^
CD4 count			
mean (cells/μL ± SD)		380.7 ± 235.4	
CD4 < 200 cells/μL, *n* (%)		14.0 (20.0)	
CD4 200–350 cells/μL, *n* (%)		20.0 (28.6)	
CD4 350–500 cells/μL, *n* (%)		21.0 (30.0)	
CD4 ≥ 500 cells/μL, *n* (%)		15.0 (21.4)	
Viral load			
Median, copies/mL (IQR)		4230 (903–91143)	
< 150 copies/mL, *n* (%)		40 (57.1)	
150–1000 copies/mL, *n* (%)		8 (11.4)	
> 1000 copies/mL, *n* (%)		22 (31.4)	
HAART history at enrollment			
< 6 months, *n* (%)		36 (51.4)	
6–12 months, *n* (%)		9 (12.9)	
> 1 year, *n* (%)		25 (35.7)	
HAART drug regimens			
3TC/EFV/ABC, *n* (%)		59 (84.3)	
3TC/EFV/AZT, *n* (%)		2 (2.9)	
3TC/EFV/D4T, *n* (%)		3 (4.3)	
3TC/NVP/ABC, *n* (%)		3 (4.3)	
3TC/Alluvia/ABC, *n* (%)		1 (1.43)	
3TC/Aluvia/AZT, *n* (%)		1 (1.43)	

*SLE* systemic lupus erythematosus, *eGFR* estimated glomerular filtration rate (MDRD equation), *HAART* highly active anti-retroviral therapy, *ESR* erythrocyte sedimentation rate, *CD* cluster of differentiation, *3TC* Lamivudine, *EFV* Efavirenz, *ABC* Abacavir, *AZT* Zidovudine, *D4T* Stavudine, *NVP* Nevirapine, *CRP* C-reactive protein, *HIV* human immunodeficiency virus

^a^Student’s *t*-test, ^b^Pearson’s *χ* ^2^ test, ^c^Fisher’s exact test, ^d^Wilcoxon-Mann-Whitney test

^e^South African Township refers to underdeveloped urban areas created under apartheid for non-white residents

### Study end points

After 18 months, 54.3% (38/70) of the HIV-negative cohort and 28.6% (20/70) of the HIV-positive cohort were alive with a patent catheter (*P* = 0.002). Technique failure occurred in 24.3% (17/70) of the HIV-negative cohort and 27.1% (19/70) of the HIV-positive cohort (*P* = 0.699), whereas 18.8% (13/70) and 40.0% (28/70), respectively, died (*P* = 0.005). One HIV-negative cohort participant and two HIV-positive participants had their Tenckhoff catheters removed due to improved renal function. One HIV-negative participant underwent kidney transplantation from a live related donor, and one HIV-positive participant left the study to undergo private hemodialysis and was lost to follow-up.

### Peritonitis episodes

There were 54 peritonitis episodes observed in 44.3% (31/70) of the HIV-negative cohort and 94 episodes in 65.7% (46/70) of the HIV-positive cohort during the follow-up period (*P* = 0.011). Fifty-one percent (36/70) of the HIV-positive cohort participants had one or more peritonitis episodes in the first 180 days following Tenckhoff catheter insertion compared to 24.3% (17/70) in the HIV-negative cohort (*P* = 0.002). Nine percent (5/54) of peritonitis episodes in the HIV-negative cohort and 13.8% (13/94) in the HIV-positive cohort were *relapse* episodes (*P* = 0.602). Eleven percent (6/54) of peritonitis episodes in the HIV-negative cohort and 7.4% (7/94) in the HIV-positive cohort were *repeat* episodes with the same organism (*P* = 0.448), while 18.5% (10/54) in the HIV-negative cohort and 23.4% (22/94) in the HIV-positive cohort were *repeat* episodes with a different organism (*P* = 0.487) (Table 2).

**Table 2:** Peritonitis outcomes at 18 months

	HIV-Negative	HIV-Positive	*P* value
Peritonitis episodes, *n* (*n* excluding *relapse*)^a^	54 (49)	94 (81)	0.001^b^
Participants with a peritonitis episode at 180 days^e^	24.3% (17/70)	51.4% (36/70)	0.001
Participants with a peritonitis episode at 1 year^e^	38.6% (27/70)	60.0% (42/70)	0.011
Participants with a peritonitis episode at 18 months^e^	44.3% (31/70)	65.7% (46/70)	0.011
Multiple peritonitis episodes			
Time between peritonitis episodes (days), median (IQR)	75 (39–107)	55.5 (34.5–115.5)	0.503^d^
*Relapse*	9.2% (5/54)	13.8% (13/94)	0.602^c^
*Recurrent*	3.7% (2/54)	6.4% (6/94)	0.711^c^
*Repeat* (Same organism)	11.1% (6/54)	7.4% (7/94)	0.448^b^
*Repeat* (Different organism)	18.5% (10/54)	23.4% (22/94)	0.487^b^
Time - Tenckhoff catheter insertion to peritonitis episode			
Median, days (IQR)	184.5 (98–370)	144.5 (63–296)	0.124^d^
Within 180 days	50.0% (27/54)	56.4% (53/94)	0.453^b^
Between 180 – 365 days	24.1% (13/54)	27.7% (26/94)	0.634^b^
Between 365 – 550 days	25.9% (14/54)	16.0% (15/94)	0.141^b^
PD WCC (cells/μl), median (IQR)	1073 (360–2690)	979 (360–2370)	0.927^d^
Outpatient treatment	33.3% (18/54)	40.4% (38/94)	0.392^b^
Inpatient treatment	66.7% (36/54)	59.6% (56/94)	0.392^b^
Inpatient stay (days), median (IQR)	9 (7–12)	8 (7–14.5)	0.574^d^
Peritonitis episode outcomes			
CAPD continuation	70.4% (38/54)	78.7% (74/94)	0.254^b^
Catheter removal	25.9% (14/54)	17.0% (16/94)	0.195^b^
Mortality	3.7% (2/54)	4.3% (4/94)	1.000^c^

*IQR* interquartile range, *PD* peritoneal dialysis, *WCC* white blood cell count, *CAPD* continuous ambulatory peritoneal dialysis, *HIV* human immunodeficiency virus

^a^Peritonitis episode count excluding peritonitis relapse^, b^Pearson’s *χ* ^2^ test, ^c^Fisher’s exact test

^d^Wilcoxon rank-sum (Mann-Whitney) test, ^e^Peritonitis experience – documented 1 or more of peritonitis episodes

The HIV-negative cohort had a higher proportion of Gram-negative peritonitis episodes (44.4%, 24/54) compared to the HIV-positive cohort (27.7%, 26/94) (*P* = 0.038). Culture-negative results were seen in 18.5% (10/54) of HIV-negative and 28.7% (27/94) of HIV-positive cohort episodes (*P* = 0.17) (Table 3). The majority of the peritonitis episodes, 70.4% (38/54) in the HIV-negative cohort and 78.7% (74/94) in the HIV-positive cohort, were successfully treated without discontinuation of CAPD. Peritonitis resulted in catheter removal in 25.9% (14/54) of HIV-negative cases and 17.0% (16/94) of HIV-positive cases accounting for 82.4% (14/17) and 84.2% (16/19) of all technique failures in each cohort, respectively. A small proportion of peritonitis episodes (3.7% (2/54) in the HIV-negative group and 4.3% (4/94) in the HIV-positive group) ended in mortality during observed admissions, accounting for 15.4% (2/13) and 14.3% (4/28) of all deaths in each cohort respectively (Table 2).

**Table 3:** Peritonitis episode culture results

	HIV-Negative	HIV-Positive	*P* value
All peritonitis episodes			
Gram-positive	33.3% (18/54)	36.2% (34/94)	0.728^a^
*Staphylococcus aureus*	16.7% (9/54)	7.4% (7/94)	0.082^a^
Coagulase-negative staphylococcus	16.7% (9/54)	23.4% (22/94)	0.332^a^
Other gram-positive bacteria	0.0% (0/54)	5.3% (5/94)	0.159^b^
Gram-negative	44.4% (24/54)	27.7% (26/94)	0.038^a^
Pseudomonas species	13.0% (7/54)	6.4% (6/94)	0.173^a^
*Klebsiella pneumoniae*	5.6% (3/54)	4.3% (4/94)	0.706^b^
Acinetobacter species	3.7% (2/54)	10.6% (10/94)	0.212^b^
Other gram-negative bacteria	22.2% (12/54)	6.4% (6/94)	0.005^a^
Mixed organisms	0.0% (0/54)	1.1% (1/94)	1.000^b^
Fungal peritonitis	3.7% (2/54)	5.3% (5/94)	1.000^b^
*Mycobacterium tuberculosis*	0.0% (0/54)	1.1% (1/94)	1.000^b^
Culture-negative	18.5% (10/54)	28.7% (27/94)	0.168^a^
Peritonitis *relapse* episodes			
Coagulase-negative staphylococcus	1.8% (1/54)	3.2% (3/94)	1.000^b^
Other gram-positive bacteria	0.0% (0/54)	1.1% (1/94)	1.000^b^
Pseudomonas species	1.8% (1/54)	3.2% (3/94)	1.000^b^
Other gram-negative bacteria	1.8% (1/54)	1.1% (1/94)	1.000^b^
Culture-negative	3.7% (2/54)^c^	4.3% (4/94)^d^	1.000^b^
*Mycobacterium tuberculosis*	0.0% (0/54)	1.1% (1/94)	1.000^b^
Peritonitis-associated catheter removal			
*Staphylococcus aureus*	1.8% (1/54)	0	0.365^b^
Pseudomonas species	9.3% (5/54)	1.1% (1/94)	0.025^b^
Acinetobacter species	1.8% (1/54)	6.4% (6/94)	0.423^b^
Other gram-negative bacteria	5.6% (3/54)	1.1% (1/94)	0.138^b^
Culture-negative	3.7% (2/54)	3.2% (3/94)	1.000^b^
Candida species	3.7% (2/54)	4.2% (4/94)	1.000^b^
*Mycobacterium tuberculosis*	0	1.1% (1/94)^e^	1.000^b^
Peritonitis-associated mortality			
Coagulase-negative staphylococcus	1.8% (1/54)	1.1% (1/94)	1.000^b^
*Escherichia coli*	1.8% (1/54)	1.1% (1/94)	1.000^b^
Fungal Peritonitis	0	1.1% (1/94)	1.000^b^
Culture-negative	0	1.1% (1/94)	1.000^b^

*HIV* human immunodeficiency virus

^a^Pearson’s *χ* ^2^ test, ^b^Fisher’s exact test

^c^One relapse episode first presented as *Staphylococcus aureus* peritonitis then relapsed as culture-negative peritonitis

^d^Two relapse episodes first presented as *Pseudomonas aeruginosa* peritonitis then relapsed as culture-negative peritonitis

^e^First presented as culture-negative peritonitis then relapsed as *Mycobacterium tuberculosis*

### Peritonitis rates and proportional hazard analysis

**Fig. 1:**
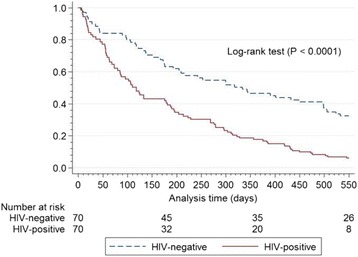
Kaplan-Meier survival estimates for peritonitis episodes excluding relapses censored for mortality, catheter loss, and loss to follow-up HIV = human immunodeficiency virus

Overall peritonitis rates, excluding *relapse* episodes, were 0.765 (HIV-negative cohort) and 1.855 (HIV-positive cohort) episodes/person-years (1/15.7 months and 1/6.5 months, respectively), with a Cox univariate proportional hazard ratio of 2.41 (95% confidence interval (CI), 1.69–3.45, *P* < 0.001) associated with HIV infection. Kaplan-Meier peritonitis-free survival rates were 6.0% (HIV-positive cohort) and 32.3% (HIV-negative cohort) at 18 months (*P* < 0.001) (Fig. 1). When the baseline CD4 count was below 200 cells/μL, the peritonitis rate rose to 3.690 episodes/person-years (HR 4.54, 95% CI 2.35–8.76, *P* < 0.001), while a baseline CD4 count above 350 cells/μL was associated with a peritonitis rate of 1.599 episodes/person-years (HR 2.10, CI 1.39–3.15, *P* = 0.001). The peritonitis *relapse* rate was 0.078 (HIV-negative cohort) and 0.298 (HIV-positive cohort) episodes/person-years (HR 3.88, CI 1.37–10.94, *P* = 0.01) (Table 4).

**Table 4:** Incidence rates and Cox proportional hazard univariate analysis

Incidence rates per person-year	HIV-Negative	HIV-Positive	Hazard ratio (95% Conf. Interval)	*P* value
All-cause peritonitis^a^	0.765	1.855	HR 2.41 (1.69–3.45)	<0.001
Baseline CD4 count				
< 200 cells/μL		3.690	HR 4.54 (2.35–8.76)	<0.001
200–350 cells/μL		1.940	HR 2.61 (1.60–4.24)	<0.001
> 350 cells/μL		1.599	HR 2.10 (1.39–3.15)	0.001
Gram-positive peritonitis	0.262	0.675	HR 2.59 (1.46–4.60)	0.001
Gram-negative peritonitis	0.353	0.512	HR 1.40 (0.80–2.44)	0.236
Culture-negative peritonitis	0.150	0.560	HR 3.64 (1.75–7.54)	0.001
Fungal peritonitis	0.028	0.089	HR 3.25 (0.63–16.79)	0.159
Peritonitis *relapse*	0.078	0.298	HR 3.88 (1.37–10.94)	0.010
Baseline CD4 count				
< 200 cells/μL		0.615	HR 10.60 (1.95–57.56)	0.006^b^
200–350 cells/μL		0.698	HR 8.82 (2.90–26.90)	<0.001^b^
≥ 350 cells/μL		0.073	HR 0.97 (0.19–5.00)	0.969^b^
Peritonitis *recurrence*	0.031	0.137	HR 4.62 (0.92–23.21)	0.063
Peritonitis *repeat* (same organism)	0.094	0.160	HR 1.81 (0.60–5.42)	0.289
Peritonitis *repeat* (different organism)	0.156	0.504	HR 3.81 (1.80–8.09)	<0.001
Multiple peritonitis^a^	0.281	0.802	HR 3.22 (1.82–5.71)	<0.001
Baseline CD4 count				
< 200 cells/μL		1.230	HR 8.41 (2.71–26.08)	<0.001^b^
200–350 cells/μL		0.931	HR 3.90 (1.86–8.18)	<0.001^b^
> 350 cells/μL		0.690	HR 2.64 (1.38–5.04)	0.003^b^
Peritonitis hospital admissions	0.815	1.814	HR 2.19 (1.44–3.35)	<0.001
Peritonitis technique failure^c^	0.195	0.285	HR 1.43 (0.69–2.93)	0.335
All-cause technique failure^d^	0.237	0.338	HR 1.42 (0.73–2.73)	0.299
Peritonitis mortality	0.028	0.071	HR 2.67 (0.49–14.60)	0.258
All-cause mortality	0.181	0.498	HR 2.53 (1.31–4.90)	0.006

*HR* hazard ratio, *CD* cluster of differentiation, *HIV* human immunodeficiency virus

^a^ Excluding peritonitis relapse episodes; ^b^ HIV-positive sub-groups compared to the HIV-negative cohort; ^c^ Peritonitis technique failure - catheter removal due to peritonitis; ^d^ Technique failure - catheter removal due to catheter malfunction or infection

On multivariable analysis, HIV (HR 1.84, 95% CI 1.07–3.16, *P* = 0.03), diabetes, and a baseline CD4 count less than 200 cells/μL were found to be independent predictors of peritonitis (Table 5).

**Table 5:** Cox proportional hazard univariate and multivariate analyses: risk factors vs. peritonitis and technique failure

	Univariate Cox proportional hazards		Multivariable Cox proportional hazards	
Variable	Hazard ratio (95% Conf. Interval)	*P* value	Hazard ratio (95% Conf. Interval)	*P* value
Peritonitis^d^				
HIV	2.41 (1.69–3.45)	<0.001	1.84 (1.07–3.16)	0.027
Race	0.19 (0.06–0.57	0.003	0.54 (0.05–5.66)	0.607
Catheter insertion method^a^	1.86 (1.28–2.71)	0.001	0.63 (0.28–1.42)	0.269
Catheter insertion site^b^	2.20 (1.45–3.33	<0.001	2.17 (0.84–5.58)	0.108
Diabetes	2.22 (1.35–3.66)	0.002	2.09 (1.09–4.03)	0.027
BMI	1.04 (1.00–1.08	0.033	1.01 (0.95–1.09)	0.720
Waist circumference	1.02 (1.00–1.04)	0.017	1.03 (0.99–1.06)	0.151
Baseline hemoglobin	0.85 (0.77–0.94)	0.002	1.02 (0.88–1.19)	0.786
Baseline albumin	0.95 (0.93–0.98)	0.001	0.98 (0.94–1.02)	0.298
Baseline CRP	1.00 (1.00–1.01)	0.004	1.00 (1.00–1.00)	0.652
Baseline CD4 count (cells/µL)				
HIV-negative	Reference			
CD4 < 200	4.54 (2.35–8.76)	<0.001	3.28 (1.42–7.61)	0.006
CD4 200–350	2.61 (1.6–4.24)	<0.001	1.18 (0.66–2.12)	0.577
CD4 ≥ 350	2.10 (1.39–3.15)	<0.001	1.00	
Residence				
City	Reference			
Township^c^	5.94 (2.17–16.25)	0.001	5.56 (0.82–37.50)	0.078
Rural area	6.07 (2.19–16.8)	0.001	4.68 (0.70–31.07)	0.110
Technique failure^e^				
HIV	1.42 (0.73–2.73)	0.299	0.39 (0.14–1.11)	0.077
Peritonitis	9.29 (2.84–30.36)	<0.001	14.47 (2.79–75.00)	0.001
Catheter insertion site	2.33 (1.09–4.97)	0.029	2.73 (0.49–15.21)	0.252
Catheter insertion method	1.62 (0.79–3.30)	0.185	0.69 (0.13–3.71)	0.663
Average hemoglobin	0.72 (0.59–0.88)	0.001	0.75 (0.59–0.95)	0.016
Average CRP	1.02 (1.01–1.02)	<0.001	1.02 (1.01–1.03)	<0.001

*BMI* Body mass index, *CD* cluster of differentiation, *CRP* C-reactive protein, *HIV* human immunodeficiency virus

^a^Catheter insertion method- laparoscopic vs. percutaneous, ^b^Catheter insertion site - Inkosi Albert Luthuli Central Hospital vs King Edward VIII Hospital, ^c^South African Township refers to underdeveloped urban areas created under apartheid for non-white residents

^d^Adjusted for age, race, gender, smoking, diabetes, body mass index, waist circumference, baseline hemoglobin, baseline serum albumin, baseline C-reactive protein, primary residence, highest education level, employment, baseline CD4 count, Tenckhoff catheter insertion site, and Tenckhoff catheter insertion method (laparoscopic vs. percutaneous)

^e^Adjusted for HIV, peritonitis, age, gender, smoking, diabetes, body mass index, waist circumference, average hemoglobin, average C-reactive protein, average serum ferritin, primary residence, highest education level, employment, Tenckhoff catheter insertion site, and Tenckhoff catheter insertion method (laparoscopic vs. percutaneous)

### Technique failure

**Fig. 2:**
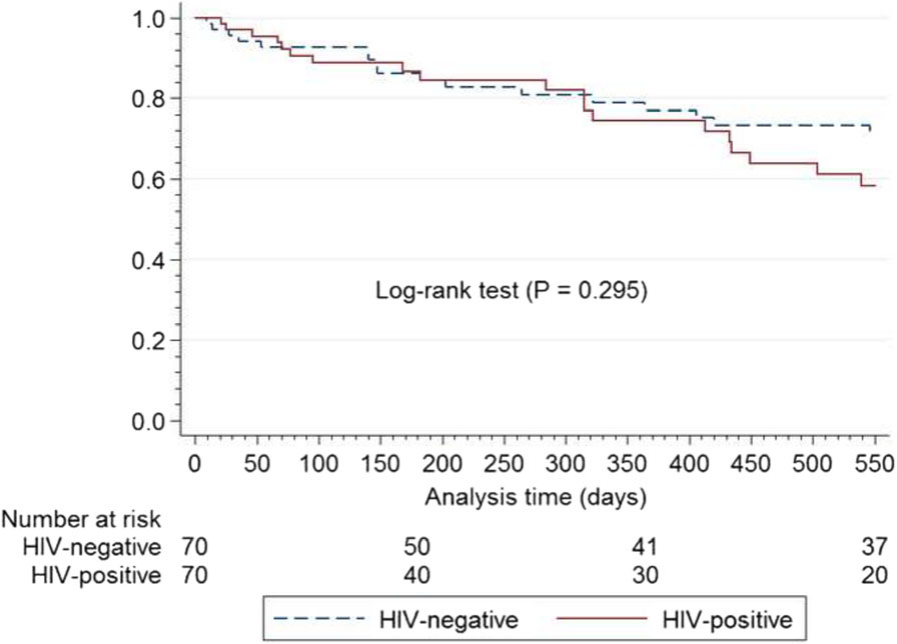
Kaplan-Meier estimates for catheter patency according to HIV status censored for mortality, loss to follow-up, and catheter removal unrelated to technique failure HIV = human immunodeficiency virus

**Fig. 3:**
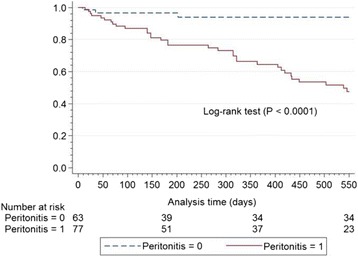
Kaplan-Meier estimates for catheter patency according to peritonitis experience (1 or more peritonitis episodes during follow-up) censored for mortality, loss to follow-up, and catheter removal unrelated to catheter failure

All-cause technique failure rates were 0.237 (HIV-negative cohort) and 0.338 (HIV-positive cohort) episodes/person-years (HR 1.42, 95% CI 0.73–2.73, *P* = 0.299). Kaplan-Meier technique survival rates at 18 months censored for death, catheter removal not related to technique failure, and loss to follow-up were 71.4% (HIV-negative cohort) and 58.2% (HIV-positive cohort), respectively (*P* = 0.295) (Fig. 2). Fifty-three percent (9/17) of technique failures in the HIV-negative cohort and 42.1% (8/19) in the HIV-positive cohort were due to gram-negative peritonitis episodes (*P* = 0.516). Fungal peritonitis was responsible for 11.8% (2/17) (HIV-negative cohort) and 21.0% (4/19) (HIV-positive cohort) of the technique failures (*P* = 0.662). Multivariable proportional hazard analysis identified peritonitis (HR 14.47, CI 2.79–75.00, *P* = 0.001), average hemoglobin concentration and average serum CRP level as independent predictors of technique failure (Table 5). Participants with one or more episodes of peritonitis during follow-up had an 18-month survival rate (Kaplan-Meier technique) of 47.5% compared to 93.9% for those who did not experience peritonitis (*P* < 0.0001) (Fig. 3).

## Discussion

This prospective cohort study evaluated the effect of HIV infection on CAPD-associated peritonitis outcomes in patients with ESRD requiring dialysis. At 18 months, HIV was associated with an increased risk (HR 2.41) of developing peritonitis with rates of 1.86 episodes/person-years compared to 0.76 episodes/person-years for HIV-negative CAPD patients. Our HIV-negative peritonitis rate was higher than the target rate of 0.67/year-at-risk advocated by the 2010 International Society for Peritoneal Dialysis (ISPD) guidelines, probably reflecting a higher intrinsic risk in our patient population which is predominantly impoverished with few available choices for alternative hemodialysis [24].

The few retrospective studies that have examined the outcomes of CAPD in HIV-infected patients have demonstrated improvements in survival and reductions in peritonitis rates associated with the use of HAART and advances in CAPD [8, 17, 26]. However, to our knowledge, our study is the first to prospectively evaluate the effects of HIV infection and duration of HAART on peritonitis outcomes among ESRD patients on CAPD. Our HIV-positive CAPD-associated peritonitis rate was much lower than the 3.9 episodes/patient-year reported over 20 years ago by Tebben et al. [17], reflecting a decreased risk associated with the greater availability of HAART over the years. Further, the authors reported a decreased peritonitis rate of 2.6 episodes/patient-year for HIV-positive patients using the Y-disconnect system, highlighting improved outcomes associated with technique enhancements. Khanna et al. [8] reported a lower peritonitis rate of 1.4 episodes/patient-year at the beginning of the HAART era; however, little information was provided on the characteristics of their HIV-positive CAPD population. Our state-sponsored renal replacement program practices a “PD first” policy directing that all dialysis-requiring ESRD patients be routinely started on CAPD. Limited hemodialysis slots are thereby reserved for those who fail CAPD or have medical contraindications to CAPD. This unselective policy determining our CAPD patient population along with the low educational levels and socioeconomic status of our patients (a majority being unemployed, living in impoverished areas, and not having completed grade 12) may have contributed to an increased intrinsic peritonitis risk [14, 27, 28].

Although HIV and diabetes were identified as independent predictors of poor peritonitis outcome, the immunologic state also modified the HIV-associated risk. A baseline CD4 count <200 cells/μL increased the hazards for peritonitis more than 4-fold compared to HIV-negative CAPD patients (3.69 episodes/person-years, HR 4.54, *P* < 0.001). This probably reflects compromised host defense mechanisms against infectious organisms at lower CD4 counts. A baseline CD4 count above 350 cells/μL was associated with a 2-fold increased hazard for peritonitis (1.60 episodes/person-years, HR 2.10, *P* = 0.001), further highlighting the inherent risk associated with HIV infection even with higher CD4 counts. The peritonitis risk was demonstrated to manifest early, as within 180 days following Tenckhoff catheter insertion half of the HIV-positive cohort had at least one documented episode of peritonitis. This risk was shown to persist, as demonstrated by the peritonitis-free survival rate of only 6.0% at 18 months.

The HIV-positive cohort showed an increased gram-positive peritonitis rate compared to the HIV-negative cohort (0.68 vs. 0.26 episodes/person-years, HR 2.59, *P* = 0.001), possibly reflecting a greater susceptibility to touch contamination-related infection due to compromised local defense mechanisms contributing to the HIV-associated peritonitis risk. This finding suggests a role for a prophylactic antibiotic strategy, particularly in the first six months following catheter insertion when the peritonitis risk is highest and more so among patients with low CD4 counts. The HIV-positive cohort also had a significantly increased culture-negative peritonitis rate compared to the HIV-negative cohort (0.56 vs. 0.15 episodes/person-years, HR 3.64, *P* = 0.001). Culture negative cases accounted for 28.7% of the HIV-positive cohort’s total peritonitis episodes; this percentage is above the 20% recommended by the 2010 ISPD guidelines. This finding could indicate a higher prevalence of fastidious organisms and mycobacteria in this group [29, 30].

Peritonitis was shown to be the predominant cause of technique failure in both cohorts and was further identified as an independent predictor of this outcome. Although HIV was associated with an increased risk of peritonitis as well as an increased risk for subsequent episodes, it was not shown to significantly influence all-cause technique failure rates (HR 1.42, *P* = 0.299) or peritonitis-associated technique failure rates (HR 1.43, *P* = 0.335). This inconsistency may be partially explained by the significantly higher proportion of gram-negative peritonitis episodes documented in the HIV-negative cohort (44.4 vs. 27.7%, *P* = 0.038); gram-negative organisms were also the major causative organism group for technique failures in both cohorts (52.9% and 42.1%, respectively). It may be that HIV infection does not increase the risk of catheter-threatening peritonitis in the first 18 months following insertion but instead increases the risk for treatable peritonitis episodes. However, increased *relapses* and multiple episodes raise concern about the long-term risk of technique failure. Further, the disproportionately higher mortality rate in the HIV-positive cohort contributed to a dropout rate of 44.3% compared to 21.4% in the HIV-negative cohort; this may have introduced bias, resulting in a lower apparent rate of technique failure in the HIV-positive cohort.

The major limitation of our study is that it is a single-center observational study, which inherently limits causation inferences that can be drawn from observed associations. Statistical power, particularly relating to technique failure outcomes, was limited by the relatively small sample size and short follow-up period. The matching strategy of restricting the inclusion age to 18–60 years may limit the generalizability of our results to only this age group. This matching strategy was employed to minimize age as a confounding factor, as HIV-positive CAPD populations are typically younger than their HIV-negative counterparts [8, 17]. More studies are needed to assess outcomes of renal replacement modalities in various HIV-positive ESRD populations.

## Conclusions

Our study indicates that HIV infection can adversely influence CAPD-associated peritonitis rates, and this association is further modified by the immunological state of the infected patient. The peritonitis risk attributable to HIV infection manifests early in the course of CAPD treatment and increases the risk for subsequent episodes, but it was not shown to result in increased technique failure rates at 18 months. Early detection of CKD and HIV with the initiation of HAART before significant immunological compromise and careful management of comorbid conditions can help minimize the risk. Prophylactic antibiotics should be considered and investigated as possible strategies to help improve peritonitis outcomes.

## Abbreviations

CAPD: Continuous ambulatory peritoneal dialysis
CD4: Cluster of differentiation 4
CI: Confidence interval
CKD: Chronic kidney disease
CRP: C-reactive protein
ELISA: Enzyme-linked immunosorbent assay
ESRD: End-stage renal disease
HAART: Highly active antiretroviral therapy
HIV: Human immunodeficiency virus
HR: Hazard ratio
IALCH: Inkosi Albert Luthuli Central Hospital
IQR: Interquartile range
KEH: King Edward VIII Hospital
NHLS: South African National Health Laboratory Service
PD: Peritoneal dialysis
WCC: White blood cell count
